# The predictive value of fresh embryo transfer pregnancy results on frozen embryo transfer outcomes: a cohort study

**DOI:** 10.3389/fendo.2026.1846758

**Published:** 2026-06-16

**Authors:** Anqi Ren, Xia Xue, Juanzi Shi, Tingting He, Wenhao Shi

**Affiliations:** Assisted Reproduction Center, Northwest Women’s and Children’s Hospital, Xi’an, China

**Keywords:** fresh embryo transfer, frozen embryo transfer, live birth, miscarriage, non-pregnancy

## Abstract

**Objective:**

To investigate whether the outcome of a fresh embryo transfer can predict the results of subsequent frozen embryo transfers (FET) in patients undergoing single blastocyst transfer.

**Design:**

Retrospective cohort study.

**Setting:**

Tertiary-care academic medical center.

**Patients:**

This retrospective study involved a total of 6,491 women who underwent single blastocyst transfer in FET cycles from January 2016 to June 2024. Women were categorized into four groups based on their fresh transfer outcomes: the non-pregnancy group (n=3,711), biochemical pregnancy group (n=701), miscarriage group (n=1,066), and live birth group (n=1,013).

**Intervention(s):**

None.

**Main outcome measure(s):**

Live birth rates.

**Result(s):**

Compared with the fresh-cycle live birth group, patients in fresh-cycle non-pregnancy, biochemical pregnancy, and miscarriage groups exhibited significantly lower chance of subsequent biochemical pregnancy (adjusted odds ratio [aOR]=0.58, 95% confidence interval [CI]: 0.50–0.68; aOR=0.70, 95% CI: 0.56–0.87; aOR=0.72, 95% CI: 0.60–0.88, respectively), clinical pregnancy (aOR=0.57, 95% CI: 0.49–0.66; aOR=0.65, 95% CI: 0.53–0.80; aOR=0.71, 95% CI: 0.59–0.85, respectively), and live birth (aOR=0.59, 95% CI: 0.51–0.68; aOR=0.71, 95% CI: 0.58–0.86; aOR=0.65, 95% CI: 0.54–0.77, respectively). However, no significant differences were observed in the rates of miscarriage or ectopic pregnancy among the four groups (P>0.05 for all). Further subgroup comparison demonstrated that compared with fresh-cycle non-pregnancy group, patients with prior fresh-cycle miscarriage had significantly higher rate biochemical pregnancy (aOR=1.12, 95% CI: 1.04–1.20), clinical pregnancy (aOR=1.12, 95% CI: 1.05–1.21), and miscarriage (aOR=1.14, 95% CI: 1.03–1.26), whereas the rates of ectopic pregnancy and live birth were remained comparable between the two groups (P>0.05 for all).

**Conclusion(s):**

In conclusion, compared with patients who achieved live birth in the fresh cycle, those in the non-pregnancy, biochemical pregnancy, and miscarriage groups had significantly lower chances of achieving biochemical pregnancy, clinical pregnancy, and live birth in subsequent FET cycles. These findings suggest that the outcome of fresh embryo transfer can serve as a potential predictive indicator for subsequent FET pregnancy prognosis, providing valuable clinical guidance for individualized counseling and management of patients undergoing assisted reproductive technology (ART) treatment.

## 1 Introduction

Since the birth of Louise Brown in 1978, more than 10 million babies have been conceived through *in vitro* fertilization (IVF) and intracytoplasmic sperm injection (ICSI) (1, 2). Assisted reproductive technology (ART) traditionally involves two primary approaches: fresh embryo transfer and frozen embryo transfer (FET) (3). Fresh embryo transfer was performed immediately after ovarian hyperstimulation, followed by FET in subsequent cycles when enough embryos are available (4). Therefore, the relationship between fresh ET outcomes and subsequent FET success was of significant importance.

However, up to now, only limited studies have evaluated the predictive value of fresh embryo transfer outcomes on subsequent FET results. Bushaqer NJ and El-Toukhy et al. demonstrated that successful pregnancy in fresh cycles serves as a positive prognostic indicator for subsequent FET cycle outcomes (5, 6). Conversely, evidence from another study revealed comparable live birth rates between patients with prior miscarriage and those without any prior pregnancy (32.3% vs. 33.3%) (7). A failed IVF-ET cycle not only subjects patients to considerable psychosocial stress from family and society but also imposes significant time and financial burdens (8). Consequently, patients are highly concerned about the underlying causes of implantation failure and the probability of achieving a successful pregnancy in subsequent embryo transfers (9).

Therefore, the aim of this study is to further assess the influence of fresh embryo transfer outcomes on subsequent FET results in patients undergoing single blastocyst transfer.

## 2 Methods

### 2.1 Study design and participants

This retrospective cohort study was conducted at the Center for Assisted Reproductive Technology of Northwest Women’s and Children’s Hospital from January 2016 to June 2024. We only included patients who underwent their first FET cycle following a fresh embryo transfer. Women were excluded if they met any of the following criterion: (1) oocyte donation cycles; (2) women with uterine abnormalities (uterine malformations, endometrial polyps, or submucosal myomas); (3) receiving transfer of more than one blastocyst; (4) undergoing embryo transfer; and (5) cycles with incomplete or missing data. Women who underwent preimplantation genetic testing (PGT) were also excluded in our study. Ultimately, this study included 6,491 women who underwent single blastocyst transfer, and were further stratified into four groups based on their fresh transfer outcomes: the non-pregnancy group (n=3,711), biochemical pregnancy group (n=701), miscarriage group (n=1,066), and live birth group (n=1,013). This study received ethical approval from the Ethics Committee of the Northwest Women’s and Children’s Hospital (number 2025-072-01). Given the retrospective nature of our study, the requirement for obtaining informed consent from participants was formally waived.

### 2.2 Ovarian stimulation and embryo culture protocols

All patients received individualized controlled ovarian hyperstimulation (COH) protocols based on maternal age, maternal body mass index (BMI), antral follicle count (AFC), and basal follicle stimulating hormone (FSH) levels. Follicular development was monitored through serial serum estradiol measurements and transvaginal ultrasonography. When at least 2 follicles reached >17 mm mean diameter, ovulation was triggered with human chorionic gonadotropin (HCG, 5,000–10,000 IU; Merck Serono, Germany). Oocyte retrieval was conducted 36 hours post-trigger, followed by IVF or ICSI based on semen parameters. Fertilization was assessed 16–18 hours post-insemination. Embryos were cultured sequentially in G1-plus medium (Vitrolife, Sweden) to cleavage stage (Day 3), then transferred to G2-plus medium until blastocyst stage (Day 5/6). For patients meeting fresh embryo transfer criteria, optimal-quality embryo was transferred. All surplus viable blastocysts were cryopreserved using vitrification for potential subsequent frozen embryo transfer cycles.

### 2.3 Endometrial preparation and luteal support

Endometrial preparation protocols were individualized based on the characteristics of the patients. Women with regular menstrual cycles underwent natural cycle (NC) monitoring through transvaginal ultrasound and serum LH measurements, with blastocyst transfer performed 5 days post-ovulation. For patients with irregular cycles or those requiring scheduling flexibility, either hormone replacement therapy (HRT) or gonadotropin-releasing hormone (GnRH) agonist combined with HRT (GnRH agonist-HRT) protocols were implemented, beginning oral estradiol administration on menstrual cycle day 5. Once EMT reached ≥7 mm with serum progesterone levels <1.5 ng/mL, progesterone supplementation was initiated. Blastocyst transfer was performed on the 6th day after progesterone administration. All patients received luteal support until 10 weeks of gestation in cases of successful pregnancy, with outcomes systematically documented in the electronic medical record system trained proficient nurses (10).

### 2.4 Outcome measures

Live birth was defined as the delivery of a viable infant ≥24 weeks of gestation. Biochemical pregnancy was confirmed by a serum β-hCG level > 20 IU/L, typically measured 12 days after blastocyst transfer. Clinical pregnancy was characterized by the presence of an intrauterine gestational sac on ultrasound at 6–8 weeks of gestation. Miscarriage was defined as the spontaneous loss of a clinical pregnancy before 24 weeks of gestation (11). Ectopic pregnancy was diagnosed based on the following standard criteria (1): identification of an extrauterine gestational sac by transvaginal ultrasound; or (2) presence of an echogenic adnexal mass with no intrauterine gestational sac, combined with elevated serum β−hCG levels and clinical manifestations consistent with ectopic pregnancy. The study data were systematically monitored and documented by trained nursing professionals, with all information meticulously recorded in the electronic medical record system. The data collection methodology has been previously described in detail (12).

### 2.5 Statistical analysis

The statistical analyses were performed using statistical packages R (v.3.4.3; The R Foundation) and SPSS software version 22.0 (SPSS Inc., Chicago, USA). Categorical variables were presented as frequencies and percentage, compared with Chi-squared test or Fisher’s exact test, as appropriate. Continuous variables were expressed as mean ± standard deviation (SD) and analyzed using one-way analysis of variance (ANOVA).

Multivariate regression analyses were performed to explore the predictive value of fresh embryo transfer pregnancy results on frozen embryo transfer outcomes. The model included the following covariates, selected based on clinical experience and studies published in recent years: maternal age, maternal BMI, infertility duration, endometrial thickness (EMT), infertility cause, fertilization type, endometrial preparation protocols, infertility type, good-quality blastocyst transfer, and year of oocyte retrieval. The results were presented as odds ratio (OR), adjusted odds ratio (aOR) and confidence interval (CI).

## 3 Results

### 3.1 Participant characteristics

**Figure 1:**
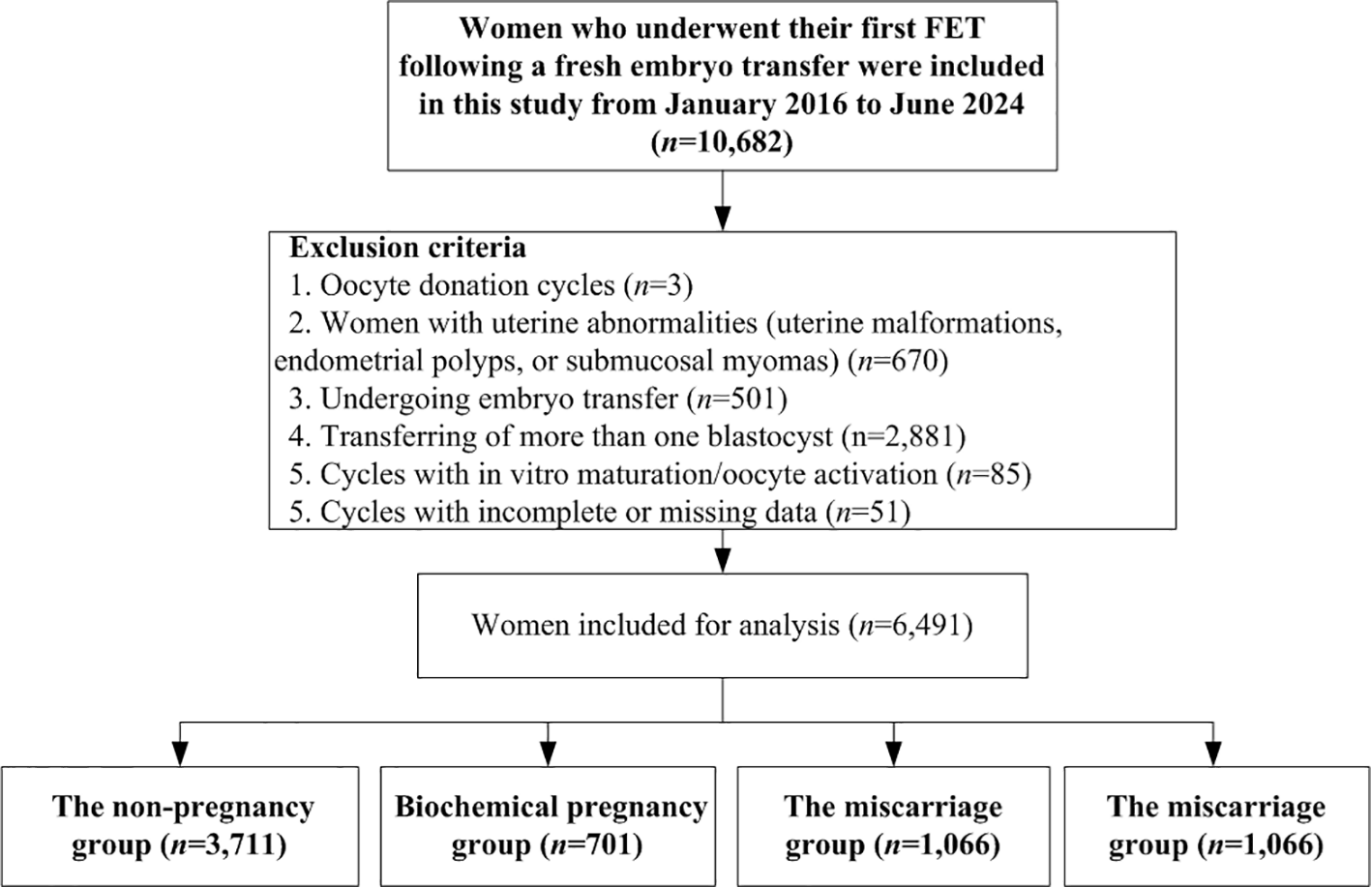
Flow chart of the study. FET frozen embryo transfer.

A total of 6,491 women who underwent their first FET following a fresh embryo transfer were screened from our database and categorized into four groups based on their fresh transfer outcomes: the non-pregnancy group (n=3,711), biochemical pregnancy group (n=701), miscarriage group (n=1,066), and live birth group (n=1,013) (**Figure 1**). The baseline characteristics of the study population were summarized in **Table 1**. Notably, significant differences were observed among the four groups in terms of maternal age, maternal BMI, infertility duration, EMT, infertility type, infertility cause, fertilization type, endometrial preparation protocols, good-quality blastocyst transfer, and year of oocyte retrieval among the four groups (P<0.05, for all).

**Table 1:** The baseline characteristics of the study population stratified by fresh embryo transfer outcome.

Characteristics	Non-pregnancy group (n=3,711)	Biochemical pregnancy group (n=701)	Miscarriage group (n=1,066)	Live birth group (n=1,013)	P-value
Maternal age (y)	31.85 ± 4.08	31.33 ± 3.96	32.21 ± 3.94	32.19 ± 3.30	<0.001^*^
Maternal BMI (kg/m2)	22.59 ± 3.33	22.66 ± 3.41	23.18 ± 3.51	22.52 ± 3.16	<0.001^*^
Infertility duration (y)	3.42 ± 2.47	3.36 ± 2.34	3.48 ± 2.41	3.09 ± 2.07	0.023^*^
EMT (mm)	10.42 ± 1.78	10.52 ± 1.74	10.41 ± 1.85	10.89 ± 1.76	<0.001^*^
Infertility type, n (%)					<0.001^*^
Primary infertility	1,793 (48.32)	366 (52.21)	564 (52.91)	629 (62.09)	
Secondary infertility	1,918 (51.68)	335 (47.79)	502 (47.09)	384 (37.91)	
Infertility cause, n (%)					<0.001^*^
Male factor	699 (18.84)	147 (20.97)	189 (17.73)	239 (23.59)	
Female factor	2,259 (60.87)	418 (59.63)	655 (61.44)	601 (59.33)	
Mixed	426 (11.48)	88 (12.55)	139 (13.04)	133 (13.13)	
Unexplained	327 (8.81)	48 (6.85)	83 (7.79)	40 (3.95)	
Fertilization type, n (%)					<0.001^*^
IVF	3,049 (82.16)	572 (81.60)	858 (80.49)	771 (76.11)	
ICSI	662 (17.84)	129 (18.40)	208 (19.51)	242 (23.89)	
Endometrial preparation protocols, n (%)					<0.001^*^
NC	1,219 (32.85)	218 (31.10)	403 (37.80)	466 (46.00)	
HRT	1,901 (51.23)	376 (53.64)	558 (52.35)	475 (46.89)	
GnRH agonist-HRT	591 (15.93)	107 (15.26)	105 (9.85)	72 (7.11)	
Good-quality blastocyst transfer, n (%)	1,687 (45.46)	333 (47.50)	561 (52.63)	457 (45.11)	<0.001^*^
Year of oocyte retrieval, n (%)					<0.001^*^
2015-2018	654 (17.62)	115 (16.41)	147 (13.79)	114 (11.25)	
2019-2021	1,784 (48.07)	338 (48.22)	444 (41.65)	403 (39.78)	
2022-2023	1,273 (34.30)	248 (35.38)	475 (44.56)	496 (48.96)	

Values are presented as mean ± standard deviation or number (percentage). *BMI* body mass index; *EMT* endometrial thickness; *IVF in vitro* fertilization; *ICSI* intracytoplasmic sperm injection; *NC* natural cycle; *HR*T hormone replacement therapy; *GnRH* gonadotropin-releasing hormone. ^*^P < 0.05 was considered statistically significant.

### 3.2 Neonatal outcomes

Pregnancy outcomes of the study population, stratified by fresh embryo transfer outcome, are presented in **Table 2**. Significant differences were observed among the four groups in terms of biochemical pregnancy, clinical pregnancy, and live birth rates (P>0.05, for all). Specifically, the live birth group had the highest rates of biochemical pregnancy (72.95%), clinical pregnancy (67.82%), and live birth (56.66%), whereas the non-pregnancy group had the lowest corresponding rates (61.22%, 54.38%, and 42.98%, respectively). However, no statistically significant differences were found among the four groups in miscarriage rate (P = 0.053) and ectopic pregnancy rate (P = 0.078).

**Table 2:** Pregnancy outcomes of the study population stratified by fresh embryo transfer outcome.

Pregnancy outcomes	Non-pregnancy group (n=3,711)	Biochemical pregnancy group (n=701)	Miscarriage group (n=1,066)	Live birth group (n=1,013)	P-value
Biochemical pregnancy, n (%)	2,272 (61.22)	465 (66.33)	711 (66.70)	739 (72.95)	<0.001^*^
Clinical pregnancy, n (%)	2,018 (54.38)	413 (58.92)	646 (60.60)	687 (67.82)	<0.001^*^
Miscarriage, n (%)	400 (10.78)	69 (9.84)	144 (13.51)	112 (11.06)	0.053
Ectopic pregnancy, n (%)	23 (0.62)	2 (0.29)	9 (0.84)	1 (0.10)	0.078
Live birth, n (%)	1,595 (42.98)	342 (48.79)	493 (46.25)	574 (56.66)	<0.001^*^

Values are presented as number (percentage). *P < 0.05 was considered statistically significant.

### 3.3 Impact of fresh cycle pregnancy outcomes on frozen embryo transfer results

Multivariable regression analysis was performed to evaluate the predictive value of fresh embryo transfer pregnancy results on frozen embryo transfer outcomes, adjusting for confounding factors including maternal age, maternal BMI, infertility duration, EMT, infertility cause, fertilization type, endometrial preparation protocol, infertility type, good-quality blastocyst transfer, and year of oocyte retrieval (**Table 3**). Compared with the fresh-cycle live birth group, patients in fresh-cycle non-pregnancy, biochemical pregnancy, and miscarriage groups exhibited significantly lower chance of subsequent biochemical pregnancy (aOR=0.58, 95% CI: 0.50–0.68; aOR=0.70, 95% CI: 0.56–0.87; aOR=0.72, 95% CI: 0.60–0.88, respectively), clinical pregnancy (aOR=0.57, 95% CI: 0.49–0.66; aOR=0.65, 95% CI: 0.53–0.80; aOR=0.71, 95% CI: 0.59–0.85, respectively), and live birth (aOR=0.59, 95% CI: 0.51–0.68; aOR=0.71, 95% CI: 0.58–0.86; aOR=0.65, 95% CI: 0.54–0.77, respectively). However, no significant differences were observed in the rates of miscarriage or ectopic pregnancy among the four groups (P>0.05 for all). We further compared the subsequent pregnancy outcomes between the miscarriage group and the non-pregnancy group (**Table 4**). Compared with fresh-cycle non-pregnancy group, patients with prior fresh-cycle miscarriage had significantly higher rate biochemical pregnancy (aOR=1.12, 95% CI: 1.04–1.20), clinical pregnancy (aOR=1.12, 95% CI: 1.05–1.21), and miscarriage (aOR=1.14, 95% CI: 1.03–1.26), whereas the rates of ectopic pregnancy and live birth were remained comparable between the two groups (P>0.05 for all).

**Table 3:** Unadjusted and adjusted associations between fresh and subsequent FET pregnancy outcomes.

Frozen embryo transfer outcomes	Group divided by fresh embryo transfer outcomes				
	Statistical Model	Live birth (ref)	Non-pregnancy	Biochemical pregnancy	Miscarriage
Biochemical pregnancy	OR (95%CI; P)	1.00; —	0.59 (0.50–0.68); <0.001*	0.73 (0.59–0.90); 0.003*	0.74 (0.62–0.90); 0.002*
	aOR (95%CI; P)	1.00; —	0.58 (0.50–0.68); <0.001*	0.70 (0.56–0.87); 0.001*	0.72 (0.60–0.88); 0.001*
Clinical pregnancy	OR (95%CI; P)	1.00; —	0.57 (0.49–0.66); <0.001*	0.68 (0.56–0.83); <0.001*	0.73 (0.61–0.87); 0.001*
	aOR (95%CI; P)	1.00; —	0.57 (0.49–0.66); <0.001*	0.65 (0.53–0.80); <0.001*	0.71 (0.59–0.85); <0.001*
Miscarriage	OR (95%CI; P)	1.00; —	0.97 (0.78–1.21); 0.801	0.88 (0.64–1.21); 0.422	1.26 (0.97–1.64); 0.089
	aOR (95%CI; P)	1.00; —	0.92 (0.73–1.15); 0.458	0.85 (0.61–1.16); 0.302	1.19 (0.91–1.55); 0.207
Ectopic pregnancy	OR (95%CI; P)	1.00; —	6.31 (0.85–46.79); 0.071	2.90 (0.26–32.00); 0.386	8.62 (1.09–68.14); 0.041*
	aOR (95%CI; P)	1.00; —	5.80 (0.77–43.42); 0.087	2.54 (0.23–28.35); 0.449	7.92 (0.99–63.07); 0.051
Live birth	OR (95%CI; P)	1.00; —	0.58 (0.50–0.66); <0.001*	0.73 (0.60–0.88); 0.001*	0.66 (0.55–0.78); <0.001*
	aOR (95%CI; P)	1.00; —	0.59 (0.51–0.68); <0.001*	0.71 (0.58–0.86); 0.001*	0.65 (0.54–0.77); <0.001*

Adjusted for maternal age, maternal BMI, infertility duration, EMT, infertility cause, fertilization type, endometrial preparation protocols, infertility type, good-quality blastocyst transfer, and year of oocyte retrieval. *OR* odds ratio; aOR adjust odds ratio; *CI* confidence interval; *BMI* body mass index; *EMT* endometrial thickness. ^*^P < 0.05 was considered statistically significant.

**Table 4:** Comparison of pregnancy outcomes between miscarriage and non-pregnancy groups.

Pregnancy outcomes	OR (95% CI)	P value	aOR (95% CI)	P value
Biochemical pregnancy, n (%)	1.13 (1.05, 1.21)	0.001	1.12 (1.04, 1.20)	0.003*
Clinical pregnancy, n (%)	1.14 (1.06, 1.22)	<0.001	1.12 (1.05, 1.21)	0.002*
Miscarriage, n (%)	1.14 (1.03, 1.26)	0.014	1.14 (1.03, 1.26)	0.014*
Ectopic pregnancy, n (%)	1.17 (0.79, 1.72)	0.43	1.15 (0.78, 1.70)	0.482
Live birth, n (%)	1.07 (1.00, 1.14)	0.058	1.06 (0.98, 1.13)	0.138

Adjusted for maternal age, maternal BMI, infertility duration, EMT, infertility cause, fertilization type, endometrial preparation protocols, infertility type, good-quality blastocyst transfer, and year of oocyte retrieval. *OR* odds ratio; aOR adjust odds ratio; *CI* confidence interval; *BMI* body mass index; *EMT* endometrial thickness. ^*^P < 0.05 was considered statistically significant.

## 4 Discussion

In this large-scale retrospective cohort study, we aimed to investigate whether the outcome of fresh embryo transfer could predict the results of subsequent FET cycles in patients undergoing single blastocyst transfer. Our results demonstrated that, compared with patients who achieved live birth in the fresh cycle, those in the non-pregnancy, biochemical pregnancy, and miscarriage groups had significantly lower chances of achieving biochemical pregnancy, clinical pregnancy, and live birth in subsequent FET cycles.

Existing studies examining the prognostic value of initial pregnancy outcomes for subsequent reproductive performance have yielded inconsistent conclusions. A study of 84 patients showed that clinical pregnancy in fresh cycles predicted significantly higher pregnancy rates in subsequent FET cycles (56.3% vs 12.1%, P<0.001) (5). However, Sneeringer et al. found that while initial IVF cycle pregnancy loss was associated with higher biochemical pregnancy rates (12.8% vs 4.1%), it did not predict live birth success in women >40 years (13). Similarly, Yang et al. reported increased early pregnancy loss (14.75% vs 7.41%) but comparable live birth rates (33.3% vs 32.3%) after prior spontaneous abortion (7). However, Turgut et al. showed comparable miscarriage rates between groups (95% CI: 0.678-12.131) (14), while another study found first-trimester pregnancy loss in initial FET cycles improved subsequent live birth rates, unlike biochemical or ectopic pregnancies which showed no significant impact (15). However, the findings of these studies may be compromised by several confounding factors. The primary limitation is the small sample sizes ranging from 84 to 404, which reduce statistical power and may affect the reliability of the conclusions (5, 13, 14). Moreover, these studies failed to adequately account for crucial variables, including differences between fresh and frozen IVF cycles, embryo quality, and the number of embryos transferred (5–7). Consequently, their ability to draw definitive conclusions was significantly limited. To address these limitations, our study specifically examined women undergoing their first FET cycle with single blastocyst transfer, evaluating the potential influence of previous fresh embryo transfer outcomes. Our findings demonstrate that prior fresh embryo transfer outcomes have predictive value for success in subsequent first FET cycles.

The findings of this study suggest that while patients with a prior miscarriage exhibit higher rates of biochemical pregnancy, clinical pregnancy, and subsequent miscarriage in their first FET cycle, their live birth rates remain comparable to those of the non-pregnancy group. This discrepancy may be explained by distinct endometrial and immunological factors influencing early pregnancy events (16, 17). Specifically, an altered endometrial immune microenvironment, characterized by impaired immune tolerance, dysregulated cytokine profile, and abnormal NK cell activity could predispose to early pregnancy loss despite successful implantation (16, 18, 19). Additionally, by focusing on FET cycles, this study minimized the confounding effects of supraphysiologic E2 levels from ovarian stimulation, providing a clearer assessment of endometrial receptivity. However, the absence of differences in live birth rates implies that once pregnancy progresses beyond the early stages, factors such as placental development and embryonic genetic fitness become more critical determinants of reproductive success (20, 21). These findings align with recent concepts of embryonic resilience and suggest that while endometrial factors may influence initial implantation success, embryonic factors become predominant determinants of ultimate reproductive outcome (22). This distinction has important clinical implications for patient counseling and may guide future research into targeted interventions for improving early pregnancy maintenance.

The present study has several notable strengths. First, we strictly included women transferred with only one blastocyst transfer in the first FET cycle following a previous failed fresh embryo transfer to ensure a homogeneous study population. Second, the consistency in clinical and laboratory protocols throughout the study period minimized potential bias. Third, we accounted for all major known confounders affecting pregnancy outcomes by incorporating them into a multivariate logistic regression model, including maternal age, infertility causes, and number of transferred embryos et al. This rigorous statistical approach strengthens the reliability of our conclusions.

However, this study has several limitations. First, the retrospective nature of the analysis inherently limits the control over data collection processes and may introduce selection bias, despite our rigorous adjustment for known confounders through multivariate modeling. Second, the single-center design, while ensuring consistency in clinical protocols, may restrict the external validity of our findings as patient demographics and laboratory practices vary across different fertility centers. Third, although multivariate logistic regression was applied to fully adjust for all baseline confounding factors, such adjustment cannot completely eliminate inherent baseline bias, which may attenuate the predictive performance of fresh cycle outcomes for subsequent FET pregnancy outcomes. Last but not least, given the retrospective nature of our study, detailed etiological information for individual fresh cycle failures was incomplete and difficult to uniformly stratify across all enrolled patients.

## 5 Conclusion

In conclusion, our large-scale retrospective study demonstrates that patients with fresh-cycle non-pregnancy, biochemical pregnancy, or miscarriage exhibit significantly reduced rates of biochemical pregnancy, clinical pregnancy, and live birth in subsequent FET cycles compared with those who achieve fresh-cycle live birth. Further stratified analyses indicate that individuals with prior fresh-cycle miscarriage have higher odds of subsequent biochemical pregnancy, clinical pregnancy, and miscarriage relative to patients with fresh-cycle non-pregnancy. Collectively, these findings suggest that fresh embryo transfer outcomes serve as a reliable prognostic indicator for subsequent FET performance, providing practical evidence to support individualized patient counseling and clinical risk stratification in routine ART practice. Given the limitations of this study, further well-designed prospective studies with detailed etiological stratification are warranted to validate our findings and optimize clinical strategies for improving reproductive outcomes.

## Data Availability

The raw data supporting the conclusions of this article will be made available by the authors, without undue reservation.

## Supplementary material

Supplementary Table 1 Subgroup analysis stratified by good-quality blastocyst transfer.

## References

[1] Fishel S . First *in vitro* fertilization baby-this is how it happened. Fertility Sterility. (2018) 110:5–11. doi: 10.1016/j.fertnstert.2018.03.008 29908772

[2] Tocariu R Niculae LE Niculae A Carp-Velișcu A Brătilă E . Fresh versus frozen embryo transfer in *in vitro* fertilization/intracytoplasmic sperm injection cycles: a systematic review and meta-analysis of neonatal outcomes. Med (Kaunas Lithuania). (2024) 60 (8):1373 doi: 10.3390/medicina60081373 39202656 PMC11356234

[3] Wei D Sun Y Zhao H Yan J Zhou H Gong F . Frozen versus fresh embryo transfer in women with low prognosis for *in vitro* fertilisation treatment: pragmatic, multicentre, randomised controlled trial. BMJ (Clinical Res Ed). (2025) 388:e081474. doi: 10.1136/bmj-2024-081474 39880462 PMC11778674

[4] Zaat T Zagers M Mol F Goddijn M van Wely M Mastenbroek S . Fresh versus frozen embryo transfers in assisted reproduction. Cochrane Database Systematic Rev. (2021) 2:Cd011184. doi: 10.1002/14651858.CD011184.pub3 33539543 PMC8095009

[5] Bushaqer NJ Alkhudhairy NN Alturaigi ZM Alhamad RM Mohawesh WA Alraka FE . The effect of fresh IVF cycle characteristics on frozen embryo transfer (FET) outcomes. JBRA Assisted Reprod. (2020) 24:135–42. doi: 10.5935/1518-0557.20190074 32072802 PMC7169927

[6] El-Toukhy T Kopeika JY Beebeejaun Y El Tokhy O Pundir J Khalaf Y . Impact of the outcome of fresh blastocyst transfer on the subsequent frozen-thawed blastocyst transfer cycle. Reprod Biomedicine Online. (2017) 35:536–41. doi: 10.1016/j.rbmo.2017.06.024 28754548

[7] Yang R Yang S Li R Chen X Wang H Ma C . Biochemical pregnancy and spontaneous abortion in first IVF cycles are negative predictors for subsequent cycles: an over 10,000 cases cohort study. Arch Gynecology Obstetrics. (2015) 292:453–8. doi: 10.1007/s00404-015-3639-8 25663163

[8] Ikemoto Y Kuroda K Endo M Tanaka A Sugiyama R Nakagawa K . Analysis of severe psychological stressors in women during fertility treatment: Japan-Female Employment and Mental health in Assisted reproductive technology (J-FEMA) study. Arch Gynecology Obstetrics. (2021) 304:253–61. doi: 10.1007/s00404-020-05923-6 33386414 PMC7775729

[9] Hill MJ . Recurrent implantation failure: Sapereaude. Fertility Sterility. (2021) 116:1430–1. doi: 10.1016/j.fertnstert.2021.09.030 34742557

[10] He T Shi W Xue X Shi J . Effect of vanishing twin on singleton neonatal outcomes in frozen embryo transfer cycles. Fertility Sterility. (2025) 124:506–13. doi: 10.1016/j.fertnstert.2025.05.146 40398717

[11] Bender Atik R Christiansen OB Elson J Kolte AM Lewis S Middeldorp S . ESHRE guideline: recurrent pregnancy loss. Hum Reprod Open. (2018) 2018:hoy004. doi: 10.1093/hropen/hoy004 31486805 PMC6276652

[12] Liu X Wang H Pan R Li Q Shi J Zhang S . Comparison of the method of endometrial preparation prior to frozen-thawed embryo transfer: a retrospective cohort study from 9733 cycles. Reprod Sci. (2021) 28:3155–63. doi: 10.1007/s43032-021-00603-5 33970443

[13] Sneeringer R Klipstein S Ryley DA Alper MM Reindollar RH . Pregnancy loss in the first *in vitro* fertilization cycle is not predictive of subsequent delivery in women over 40 years. Fertility Sterility. (2008) 89:364–7. doi: 10.1016/j.fertnstert.2007.02.038 17482171

[14] Turgut NE Boynukalin FK Gultomruk M Yarkiner Z Abali R Bahceci M . From live birth to live birth: a strong correlation between the outcomes of first and second frozen-thawed euploid blastocyst transfers from sibling oocytes. J Assisted Reprod Genet. (2025) 42:193–200. doi: 10.1007/s10815-024-03329-w 39562396 PMC11806171

[15] Li J Lin J Yin M Zhu Q Kuang Y . The live birth and neonatal outcomes in the subsequent pregnancy among patients with adverse pregnancy outcomes in first frozen embryo transfer cycles. Arch Gynecology Obstetrics. (2020) 302:731–40. doi: 10.1007/s00404-020-05608-0 32468163

[16] Wang J Han T Zhu X . Role of maternal-fetal immune tolerance in the establishment and maintenance of pregnancy. Chin Med J. (2024) 137:1399–406. doi: 10.1097/cm9.0000000000003114 38724467 PMC11188918

[17] Günther V Allahqoli L Deenadayal-Mettler A Maass N Mettler L Gitas G . Molecular determinants of uterine receptivity: comparison of successful implantation, recurrent miscarriage, and recurrent implantation failure. Int J Mol Sci. (2023) 24. doi: 10.3390/ijms242417616 38139443 PMC10743587

[18] Rao VA Kurian NK Rao KA . Cytokines, NK cells and regulatory T cell functions in normal pregnancy and reproductive failures. Am J Reprod Immunol (New York NY 1989). (2023) 89:e13667. doi: 10.1111/aji.13667 36480305

[19] Wang F Jia W Fan M Shao X Li Z Liu Y . Single-cell immune landscape of human recurrent miscarriage. Genomics Proteomics Bioinf. (2021) 19:208–22. doi: 10.1016/j.gpb.2020.11.002 33482359 PMC8602400

[20] Soares MJ Varberg KM Iqbal K . Hemochorial placentation: development, function, and adaptations. Biol Reprod. (2018) 99:196–211. doi: 10.1093/biolre/ioy0498 29481584 PMC6044390

[21] Woods L Perez-Garcia V Kieckbusch J Wang X DeMayo F Colucci F . Decidualisation and placentation defects are a major cause of age-related reproductive decline. Nat Commun. (2017) 8:352. doi: 10.1038/s41467-017-00308-x 28874785 PMC5585348

[22] A DAA Etrusco A Riemma G Chiantera V Laganà AS Agrifoglio V . The impact of uterine disorders on embryo implantation and early survival: from molecular insights to clinical evidence. Gynecologic Obstetric Invest. (2025) 90:383–97. doi: 10.1159/000543836 39884257

