# Supplementary material for: The predictive value of fresh embryo transfer pregnancy results on frozen embryo transfer outcomes: a cohort study
Source: Front Endocrinol (Lausanne). 2026 Jun 16;17:1846758. doi: 10.3389/fendo.2026.1846758 (PMC13314449; doi:10.3389/fendo.2026.1846758)
Supplement: Supplementary Table 1 — Subgroup analysis stratified by good-quality blastocyst transfer. [file Table1.docx]

**Supplement Table 1.** Subgroup analysis stratified by good-quality blastocyst transfer.

| **Sub-group** | **N** | **Biochemical pregnancy** | **Clinical pregnancy** | **Miscarriage** | **Ectopic pregnancy** | **Live birth** |
| --- | --- | --- | --- | --- | --- | --- |
|  |  | OR (95%CI; P) | OR (95%CI; P) | OR (95%CI; P) | OR (95%CI; P) | OR (95%CI; P) |
| **Good-quality blastocyst transfer** |  |  |  |  |  |  |
| **No** |  |  |  |  |  |  |
| Non-pregnancy group | 2,024 | 1 | 1 | 1 | 1 | 1 |
| Biochemical pregnancy group | 368 | 1.28 (1.02, 1.60) 0.036^*^ | 1.23 (0.98, 1.54) 0.069 | 1.03 (0.73, 1.47) 0.856 | 1.00 (0.22, 4.53) 1.000 | 1.23 (0.98, 1.54) 0.077 |
| Miscarriage group | 505 | 1.24 (1.01, 1.51) 0.0367* | 1.33 (1.09, 1.61) 0.0048* | 1.37 (1.03, 1.82) 0.0309 | 1.09 (0.30, 3.93) 0.8910 | 1.16 (0.95, 1.42) 0.1413 |
| Live birth group | 556 | 1.95 (1.60, 2.39) <0.001^*^ | 1.88 (1.55, 2.28) <0.001^*^ | 1.15 (0.86, 1.53) 0.349 | 0.00 (0.00, Inf) 0.99 | 1.82 (1.51, 2.20) <0.001^*^ |
| **Yes** |  |  |  |  |  |  |
| Non-pregnancy group | 1,687 | 1 | 1 | 1 | 1 | 1 |
| Biochemical pregnancy group | 333 | 1.19 (0.92, 1.55) 0.187 | 1.15 (0.90, 1.47) 0.266 | 0.76 (0.50, 1.16) 0.205 | 0.00 (0.00, Inf) 0.987 | 1.29 (1.02, 1.63) 0.037* |
| Miscarriage group | 561 | 1.21 (0.98, 1.49) 0.078 | 1.17 (0.95, 1.42) 0.133 | 1.23 (0.92, 1.64) 0.172 | 1.51 (0.56, 4.04) 0.413 | 1.04 (0.86, 1.26) 0.660 |
| Live birth group | 457 | 1.44 (1.14, 1.83) 0.003^*^ | 1.67 (1.32, 2.10) <0.001^*^ | 0.89 (0.63, 1.25) 0.496 | 0.31 (0.04, 2.36) 0.256 | 1.68 (1.36, 2.08) <0.001^*^ |

*Note*: *OR* odds ratio; *CI* confidence interval.

^*^*P* < 0.05 was considered statistically significant.
